# Evolutionary Protection of Krüppel-Like Factors 2 and 4 in the Development of the Mature Hemovascular System

**DOI:** 10.3389/fcvm.2021.645719

**Published:** 2021-05-17

**Authors:** David R. Sweet, Cherry Lam, Mukesh K. Jain

**Affiliations:** ^1^Case Cardiovascular Research Institute, Case Western Reserve University, Cleveland, OH, United States; ^2^Harrington Heart and Vascular Institute, University Hospitals Cleveland Medical Center, Cleveland, OH, United States; ^3^Department of Pathology, Case Western Reserve University, Cleveland, OH, United States; ^4^Department of Biology, New York University, New York, NY, United States

**Keywords:** macrophage, endothelial cell, Kruppel like factor, evolution, GC content, myeloid cell, inflammation

## Abstract

A properly functioning hemovascular system, consisting of circulating innate immune cells and endothelial cells (ECs), is essential in the distribution of nutrients to distant tissues while ensuring protection from invading pathogens. Professional phagocytes (e.g., macrophages) and ECs have co-evolved in vertebrates to adapt to increased physiological demands. Intercellular interactions between components of the hemovascular system facilitate numerous functions in physiology and disease in part through the utilization of shared signaling pathways and factors. Krüppel-like factors (KLFs) 2 and 4 are two such transcription factors with critical roles in both cellular compartments. Decreased expression of either factor in myeloid or endothelial cells increases susceptibility to a multitude of inflammatory diseases, underscoring the essential role for their expression in maintaining cellular quiescence. Given the close evolutionary relationship between macrophages and ECs, along with their shared utilization of KLF2 and 4, we hypothesize that KLF genes evolved in such a way that protected their expression in myeloid and endothelial cells. Within this Perspective, we review the roles of KLF2 and 4 in the hemovascular system and explore evolutionary trends in their nucleotide composition that suggest a coordinated protection that corresponds with the development of mature myeloid and endothelial systems.

## Introduction

Proper function of blood vessels and the cells that flow therein is critical in maintaining oxygen and nutrient delivery, facilitating metabolic waste removal, and ensuring immunological protection to distant organs. Constantly exposed to new equilibria with each heartbeat, endothelial cells (ECs) are exquisite sensors to changes in oxygen tension, pressure, circulating molecules, and cellular activity. As such, they dynamically respond to the systemic environment, relaying signaling messages between the circulation and neighboring tissues. Likewise, innate immune cells (e.g., myeloid cells such as neutrophils and monocytes) play a pivotal role in responding to circulating stimuli and enacting tissue-level local effects. Responding to pathogen and danger-associated molecular patterns, these cells orchestrate occasionally massive inflammatory cascades to neutralize perceived threats.

Given their individual importance, it is perhaps unsurprising that the vascular and myeloid systems do not function independent of one another. Indeed, there is a vast interplay between luminal cells of the vasculature and circulating leukocytes that allows each population to adapt to and affect the functions of the other. These signaling axes are bidirectional: myeloid-derived cues lead to altered barrier permeability, while endothelial release of chemokines cues circulating immune cells to home toward sites of infection. Additionally, ECs and myeloid cells appear to be derived from the same ancient precursors (“hemangioblasts”), further underscoring their relationship throughout evolution (1).

Such interactions between cell systems allow for constant surveillance of multiple systemic parameters and to respond to an ever-shifting equilibrium. Given the fine-tuned homeostasis of the hemovascular system, perturbations in either cell compartment lead to potentially disastrous effects. Improper activation of endothelial or myeloid cells contributes to nearly all causes of morbidity and mortality in the Western world including infection, cardiovascular disease, metabolic disorders, and neurodegenerative disease, to name a few. For example, interactions between monocytes, neutrophils, and ECs have been long-appreciated to mediate atherosclerotic disease (2), and these interactions are now understood to be critical players of thrombosis (3). Further, the COVID-19 pandemic has further underscored the relationship between immune activation and endothelial dysfunction in the form of vasculitides, blood-brain barrier permeability, and overt thrombotic disease (4–8).

Proper regulation of endothelial and myeloid quiescence, therefore, is imperative in resisting chronic inflammatory diseases and the complications of acute inflammatory activation. Among the shared molecular regulators of these processes are the Krüppel-like factors (KLFs): a family of zinc-finger transcription factors with broad functions including differentiation, metabolism, and cellular quiescence. Among ECs and myeloid cells, KLFs 2 and 4 are particularly important in promoting quiescent transcriptional programs and lost expression of either results in aberrant inflammatory activation of their respective cells. While transient downregulation of these factors is essential in acute activation (e.g., fighting infection), the resolution and subsequent maintenance of proper KLF2/4 expression is vital in preventing consequences of long-term myeloid and endothelial activation.

Mechanisms to protect the expression of critical and highly expressed genes have developed across evolution. Among them is an evolutionary predilection toward elevated G/C usage in essential protein-encoding genes. Positively impacting mRNA stability, transcription and translation levels, and DNA damage reduction, mutational bias toward GC content provides insight into genes and processes deemed evolutionarily essential. Within this Perspective, we review the growing evidence implicating KLF2/4 as critical mediators of myeloid and endothelial quiescence and explore whether these factors have been protected along evolution by gaining GC-bias. Finally, we present that case that the trend of increased KLF2/4 GC content has evolved concurrently with the emergence of a mature hemovascular system.

## KLF2 and KLF4 Regulate Vascular Homeostasis and Disease

The importance of KLFs in endothelial biology has been appreciated for over two decades (9, 10). Initial studies investigating mice with targeted deletion of KLF2 resulted in embryonic lethality due to vasculogenesis defects (11, 12). This led to the investigation into whether KLF2 serves as an essential regulator of endothelial and, consequently, systemic survival. After confirming expression KLF2 in murine and human ECs, investigators demonstrated that KLF2 expression is enhanced during endothelial homeostasis (e.g., laminar flow) and is reduced as the result of turbulent flow and with cytokine stimulation (13, 14). Mechanistically, KLF2 promotes endothelial health through a variety of target genes. As an anti-inflammatory transcription factor, KLF2 suppresses inflammatory transcription in part through its competition against NFkB for critical transcriptional co-factors (14–17). KLF2 also promotes transcription of anti-thrombotic genes, further lending to its role as a vasoprotective transcription factor (16, 18–21). Altogether, endothelial KLF2 promotes quiescence and integrity through the coordination of multiple transcriptional networks (22–24).

Similar to its close family member, KLF4 is equally as important in maintaining endothelial health. Also induced by laminar sheer stress, KLF4 promotes anti-inflammatory, and anti-thrombotic gene expression (25–27). Further, KLF4 promotes endothelial quiescence throughout a vascular bed via modulating gap junctional proteins and through the maintenance of proper proteostasis (28, 29). These mechanisms protect against age-associated vascular disease, atherosclerosis, and ischemic stroke.

KLF4 targets many of the same genes and functions as KLF2 in ECs. In fact, there is mounting evidence of considerable redundancy between KLF2 and 4 functions such that the presence of one factor can make up for the lost expression of the other. Endothelial deletion of both KLF2 and KLF4 is embryonic lethal (30), but remarkably, the presence of a single allele of either KLF2 or KLF4 is sufficient for life (31). Further, hemizygous loss of KLF2 leads to a compensatory increase in KLF4 expression, illustrating the redundant nature of these factors (32). Given their role in maintaining endothelial health, it is perhaps unsurprising that loss of KLF2 or KLF4 in endothelial cells is associated with increased susceptibility to vascular disease when challenged with noxious stimuli. Indeed, KLF2/4 expression decreases in numerous vascular disease states such as atherosclerosis, stroke, pulmonary artery hypertension, and age-associated loss of vasoreactivity (27, 29, 33, 34). Likewise, conditional deletion of KLF2 or KLF4 in ECs promotes vascular inflammation and disease (19, 25, 26, 35–37). Conversely, overexpression of KLF2/4 protects against endothelial disease (35, 38). Together, these studies underscore the importance of transcriptional regulators (KLFs, in particular) in establishing and maintaining a proper functioning vasculature for the health and survival of the organism.

It should be noted that a conversation about transcriptional regulation of vascular physiology would be incompletely without a addressing the important role of vascular smooth muscle cells (VSMCs). Serving as critical responders to changes in flow dynamics, VSMCs fortify the vascular unit through a coordinated phenotype switching gene program in response to various stresses. To achieve these and other functions, VSMCs utilize KLFs. In particular, KLF4 activity promotes de-differentiation of VSMCs, leading ultimately to their proliferation (39). The role of KLF2 in VSMCs is less-well-defined, however, studies have demonstrated that endothelial KLF2 is important in modulating VSMC migration in the maturing vasculature via a number of secreted factors (40, 41). While the role of KLFs in VSMC biology has been reviewed elsewhere (9), we include a brief discussion to illustrate the interconnectedness of the hemovascular system. Given the evidence of shared functions of KLF2/4 in the endothelium and in myeloid cells (see below), these cell types are of particular interest when exploring the evolution of the hemovascular system. As additional studies arise on these factors in VSMCs, however, the extent of multi-system evolution through transcription factors may be more fully realized.

## KLF-Regulation of Myeloid Cell Activation: Evolutionary Insight into Response to Acute and Chronic Inflammation

Due to their numerous interactions in homeostasis and disease, one could teleologically predict that ECs and circulating leukocytes respond to similar stimuli by using shared regulatory mechanisms. KLF2 and 4 represent one such example whose expression is decreased by noxious stimuli (e.g., infection, inflammatory metabolites, etc) in both cell populations, allowing a concerted response against threat. Understanding the functions of KLF2/4 in immune cells provides insight into the systemic response to inflammation.

Evidence over the past decade has identified a critical role for KLF2 in regulating myeloid cell activation in acute and chronic inflammatory settings. Through its negative regulation of NFkB-mediated transcription, KLF2 resists inflammatory activation in monocytes, macrophages, and neutrophils (42–45). Much like its endothelial counterpart, myeloid KLF2 levels are sensitive to inflammatory stimuli and are decreased in acute and chronic inflammatory states such as sepsis, coronary artery disease, and metabolic disease (42, 45, 46). The downstream effects of lost KLF2 expression are multitudinous. Loss of KLF2 in macrophages affects the transcription of >1,400 genes, encompassing a wide range of pathways such as metabolism, chemotaxis, and cytokine release (46). This underscores the profound regulatory effect that KLF2 has on local and systemic inflammatory states. Functionally, loss of KLF2 permits myeloid cells to more effectively respond to acute inflammatory stimuli such as bacterial infection (45). When myeloid cell activation chronically persists, however, inflammatory diseases arise. Unsurprisingly, mice with myeloid-specific deletion of KLF2 are more susceptible to diseases such as atherosclerosis, metabolic disease, neurodegeneration, and shock (32, 45–48). Protection of myeloid KLF2 expression across the lifespan and generations, therefore, is critical in the prevention of aberrant inflammation and resultant diseases.

While KLF2 serves largely as a transcriptional gatekeeper between myeloid cell quiescence and activation, KLF4 fine tunes macrophage responses and modulates macrophage polarization. Indeed, transcriptional interactions between KLF4 and STAT6 drive alternative (M2) macrophage polarization and prevent inflammatory activation (49, 50). As such, loss of KLF4 expression in macrophages leads to classical macrophage polarization (M1) with resultant inflammatory diseases (49, 51, 52). Similar to the relationship seen in ECs, KLF2 and KLF4 negatively regulate many of the same inflammatory transcriptional targets in macrophages (43, 45, 49, 53). Indeed, unpublished data from our group demonstrates that 68% of genes that are differentially regulated with loss of KLF4 are also changed with loss of KLF2 in macrophages. Additional studies are currently underway investigating the relationship between these two factors in regulating transcription of myeloid cells.

KLF4 also has a critical role in monocyte differentiation. KLF4 expression is increased as hematopoietic precursors terminally differentiate into monocytes, a mechanism that appears to be in part due to IRF8-targeting of the *Klf4* locus (54, 55). As expected, loss of KLF4 expression in precursors prevents monocytic differentiation, while forced overexpression induces this process (54, 56). Disruption of KLF4 signaling in hematopoietic lineages throughout time, therefore, would lead to potentially catastrophic consequences on the innate immune system.

## GC Content of KLF2 Has Increased Over Evolutionary Time

The available literature, as well as ongoing studies, clearly demonstrate a partnership between KLF2 and 4 in endothelial and myeloid cells. There is an important cooperativity that occurs within each of these cell populations between KLF2 and KLF4, as well as in intercellular interactions of their transcriptional targets (e.g., inflammatory products from monocytes activating ECs). Given that disrupted expression of either factor leads to inflammatory disease, it is logical that evolution would develop mechanisms to protect expression of KLF2 and KLF4, especially as mature vascular and immune systems began to arise. We explored this concept using GC content as a proxy for evolutionary selection of critically transcribed genes.

The relative composition of nucleotides of a particular genomic region dictates much more than proteins expressed. The proportion of guanine and cytosine bases (GC content) has long been appreciated to increase DNA stability as evidenced by frustrating difficulties PCR-amplifying regions with high GC content. Studies from the past 5 years have expanded on these observations and have determined more nuanced effects of GC content on transcription and mRNA stability, underscoring a potential functional driver of GC-biased gene conversion across evolution (57–60). Using KLF2 as an exemplar of GC accumulation, we are able to explore how system-level needs would drive enhanced stability of factors critical for maintaining physiological homeostasis.

**Figure 1:**
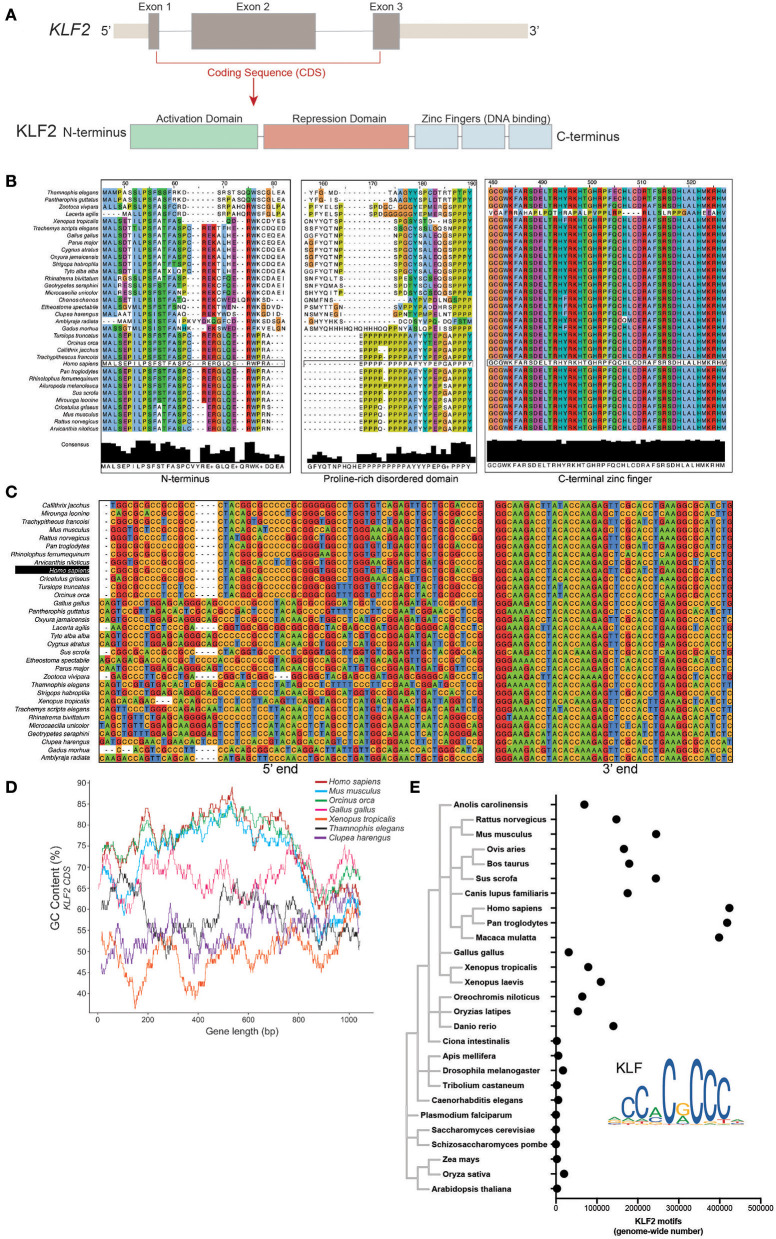
**(A)** Schematic representation of the *KLF2* gene and protein delineating exons and functional domains. **(B)** Clustal Omega amino acid alignment for KLF2 orthologs from 34 species across multiple vertebrate classes. **(C)** Clustal Omega nucleotide alignment for KLF2 orthologs. **(D)** GC content plot for representative species from different classes. GC% was calculated in a 100 bp window. **(E)** Position weight matrix analysis of KLF2 binding motif occurance in various species. Points represent number of motifs found with a significance enrichment of *p* < 0.00005.

Mammalian *KLF2* is made up of three exons encoding a ~350 amino acid (AA) protein with three main functional domains (Figure 1A). The majority of the protein is made up of highly disordered activation and repression domains responsible for co-factor interactions. Conversely, the C-terminal end of KLF2 contains three Cys2-His2 zinc finger (C2H2-ZF) regions that are structured and are highly conserved between other C2H2-ZF transcription factors (61). There is remarkable similarity of AA sequence of KLF2 orthologs within 34 species across multiple metazoan classes (Figure 1B). Despite AA conservation, nucleotide sequence alignment demonstrates considerable loss of conservation between classes (Figure 1C). Mammalian *KLF2* exhibits considerably less A/T content, especially toward the 5' end of the gene, while lower-order organisms have more conservation toward one another. Initial alignment suggests that evolutionarily recent organisms enriched GC content over time without altering the basic makeup of the KLF2 protein. Plotting GC content across the length of *KLF2* in a selection of organisms from multiple classes demonstrates that, indeed, mammals and birds have elevated GC 5' GC content, while fish, reptiles, and amphibians exhibit lower GC content (Figure 1D). To explore whether GC content elevation preferentially affects the 5' end of *KLF2*, we measured GC% within the first 300 base pairs and last 300 base pairs. These regions correspond with exon 2 (protein interaction domain) and the zinc finger DNA-binding domain, respectively, providing insight into two different important regions for KLF2 function (Figure 1A). Remarkably, *KLF2* from homo sapiens has a GC content of 73.6% within the first 300 base pairs. In contrast to the 5' end, the 3' end of *KLF2* that encodes the zinc finger regions shows little difference in GC content across classes, suggesting that these regions require less protection against mutation or negative selection over evolutionary time. Importantly, the window size used to investigate 5' vs. 3' GC bias did not appreciably affect the results, as the first 100 bp compared to the last 100 bp demonstrated a similar trend as the 300 bp window (data not shown).

As further evidence for a shift in the need for KLFs over time, we explored whether the frequency of KLF binding motifs increased in later species. Using a position weight matrix model scanner for the JASPAR-identified KLF2 motif (62, 63), we noted increased appearance of KLF motifs in later species with an extraordinary increase in primates, potentially suggesting increased utilization (Figure 1E).

## Increased GC Content in the Third Codon Position Protects the KLF2 Gene Without Affecting Protein Structure

**Figure 2:**
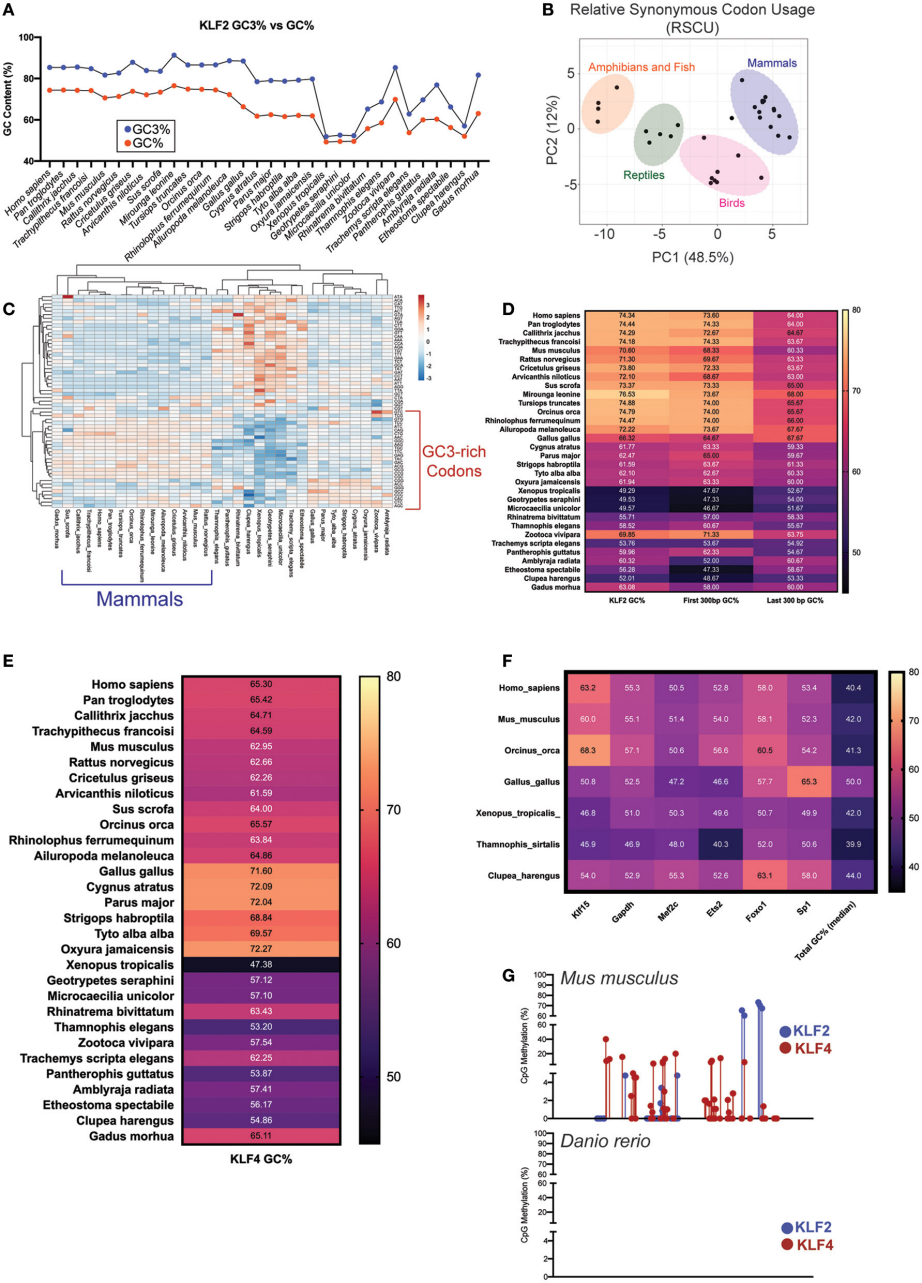
**(A)** Comparison of GC% to GC3% (G/C positioned in the 3rd base of a codon) in KLF2 across multiple species. **(B)** Principal Component Analysis (PCA) for KLF2 Relative Synonymous Codon Usage (RSCU) across numerous species. Variance largely clustered by class as delineated by color-coded ovals. **(C)** Heatmap of KLF2 RSCU for each species. Intensity of color corresponds with relative utilization of a particular nucleotide combination for a codon. Unsupervised clustering demonstrates that species within the same class or in evolutionarily close classes use similar patterns of codons. **(D)** KLF2 GC% across species demonstrating how the GC% of the first 300 bp or last 300 bp compares to whole gene GC%. **(E)** KLF4, **(F)** KLF15, GAPDH, MEF2C, ETS2, FOXO1, SP1, and total genome GC% across analyzed species. **(G)** Reduced representation bisulfite sequencing from mouse and zebrafish livers [data from Zhang et al. (76), GEO accession numbers GSM2136660, GSM2136661, GSM2136662, and GSM2136663]. Points depict %methylation at a particular CpG site along the length of the KLF2/4 gene. For all figures, GC content was calculated specifically for the coding sequences (CDS).

While overall GC content provides protection against damage (64, 65), a guanine or cytosine specifically in the third position of a codon (GC3) has its own transcriptional benefits for essential genes. The accumulation of GC3 content provides numerous benefits for protein encoding genes including higher mRNA stability, higher translation efficiency, and more methylation potential that provides mutational resistance during times of stress (66–68). Codon degeneracy allows for synonymous mutations to result in the translation of the same amino acids (69). This provides a mechanism by which GC content can increase in genes responsible for central cellular functions without dramatically altering the protein identity. While KLF2 GC content is dramatically increased in later evolving species (Figure 1), these levels are even higher in the third position of the codon (Figure 2A). Likewise, KLF2 GC3 levels appear to have increased over evolutionary time. The result of this is a shift in synonymous codon usage to those with G or C as the third position. When comparing the relative synonymous codon usage (RSCU) between major classes, there is less variation between closely related species (Figure 2B), with mammals and birds preferentially using GC3-rich codons (Figure 2C).

In addition to GC3 content increasing mRNA stability, GC content toward the 5'-end of coding genes increases mRNA transcription and protein stability (70). As previously demonstrated, the elevated GC content of KLF2 appears to be largely localized to the 5' end encoding the regulatory regions of the protein (Figure 1D). To determine the relative contribution of 5' vs. 3' ends of the KLF2 gene to the overall GC content, we compared these two regions across each species. As expected, the majority of the change across evolution occurred in the 5' end of the gene, offering another potential source of protection against mRNA and protein degradation (Figure 2D). Together, these data suggest that KLF2 experienced GC mutational bias over time in order to enhance transcriptional and translational efficiency of its gene product.

## GC Content Elevation as a Mechanism for Preserving Redundancy of KLF2 and KLF4

As previously discussed, KLF2 and KLF4 demonstrate remarkable functional overlap within myeloid and endothelial cells. Given our hypothesis that KLF2 GC content accumulation over time served to protect the establishment of a mature hemovascular system, we wanted to explore if KLF4 shared a similar evolutionary trajectory. Although the GC% of KLF4 is not as elevated as KLF2, it still possessed increased GC content compared to whole genome and exhibits increases over evolutionary time (Figure 2E). Importantly, mammalian KLF4 also utilizes GC3-rich codons more than earlier classes with differences in GC content across classes largely localized at the 5' end (data not shown). To ensure that GC content bias of KLF2/4 was not an evolutionary artifact, we calculated GC content for numerous other factors in select species (Figure 2F). First, we noted stable whole-genome median GC content around 40–50% without any trend in bias toward recent classes. Importantly, the trends we note in GC content bias in KLF2 and KLF4 are less pronounced in a more distant KLF relative, KLF15. In an unrelated, but ubiquitously utilized metabolic gene, GAPDH, we noted no GC content bias in recent species. Finally, we explored whether other transcription factors known to be critical in both endothelial and myeloid function have evolved in a similar manner. In myeloid cells, MEF2C has important implications in activation and cell death (71), while in endothelial cells, it suppresses inflammation (72). Despite its importance, MEF2C does not exhibit the same GC content increase over evolution as KLF2/4. Interestingly, MEF2C is a well-documented positive regulator of KLF2 and 4 transcription and, despite this, it appears to have not evolved in a similar pattern (73, 74). In addition to MEF2C, we explored two other hemovascular transcription factors: Ets2 and Foxo1. Neither exhibited increased GC% in recent species compared to earlier classes. Finally, Sp1, the prototypical C2H2 zinc finger transcription factor within the Sp/KLF family, does not exhibit GC bias, despite its close relationship with KLF2/4 (75).

While the functional implications of increased GC content in KLF2/4 remain to be explored, a cursory exploration about how this might affect transcriptional stability yielded interesting results. Using reduced representation bisulfite sequencing (RRBS) data from mouse and zebrafish livers (76), we noted substantial methylation within the gene body of *Klf2* and *Klf4* CpG islands in mice (Figure 2G). Interestingly, this effect was not seen in zebrafish. While there are certainly differences in CpG methylation between these species that needs to be accounted for, future work may demonstrate additional epigenetic mechanisms of evolutionary protection of KLF2 and KLF4.

## Discussion

An appropriately functioning hemovascular system is essential for proper distribution of nutrients, oxygen, and immunological surveillance throughout the body. As the size and metabolic need of organisms began to increase, there was substantial evolution of the cardiovascular and immune systems to adapt to new physiological demands. Professional phagocytes are an ancient cell type responsible for host defense found even in many invertebrate species. Throughout vertebral evolution, however, the repertoire of phagocytes (e.g., macrophages) expanded. These functions, including antigen presentation, polarization, and advanced cytokine release have continued to evolve throughout vertebrate history [reviewed in (77–80)].

Remarkably, the evolution of an advanced cardiovascular system, including endothelial cells as we understand them today, followed a similar trajectory as myeloid cells. Briefly, closed circulatory systems preceded the development of highly responsive ECs which, in turn, provided greater control of flow, responsiveness to hypoxia, and immunological defense to later vertebrate species (81). Here, we provide insights that corroborate this close evolutionary relationship between these two systems. We believe that as the functions of macrophages and ECs became more specialized and critical to the function of the entire organism, tight transcriptional control became more imperative. Therefore, we hypothesize that evolutionary pressure to protect KLF2 and 4 production caused an increase in GC content over time. Future empirical studies would be of great interest in determining how elevated GC%, GC3%, and GC-rich RSCU affect KLF2 and 4 mRNA stability, translation efficiency, and function in myeloid and endothelial cells. Additional mechanisms of evolutionary selection can also be interrogated such as the emergence of positively selected variants within these factors or GC-bias/SNV selection of regulatory regions (e.g., enhancers) known to induce the transcription of KLF2/4. Furthermore, the identification of trends in similar factors in these cell types will provide insight into how these two systems cooperatively evolved over time.

## Data Availability Statement

The raw data supporting the conclusions of this article will be made available by the authors, without undue reservation.

## Author Contributions

DS wrote the manuscript. DS and CL performed analyses. DS and MJ oversaw the project and revised the manuscript.

## Conflict of Interest

The authors declare that the research was conducted in the absence of any commercial or financial relationships that could be construed as a potential conflict of interest.
