# Supplementary figures and images for: Evolutionary Protection of Krüppel-Like Factors 2 and 4 in the Development of the Mature Hemovascular System
Source: Front Cardiovasc Med. 2021 May 17;8:645719. doi: 10.3389/fcvm.2021.645719 (PMC8165158; doi:10.3389/fcvm.2021.645719)

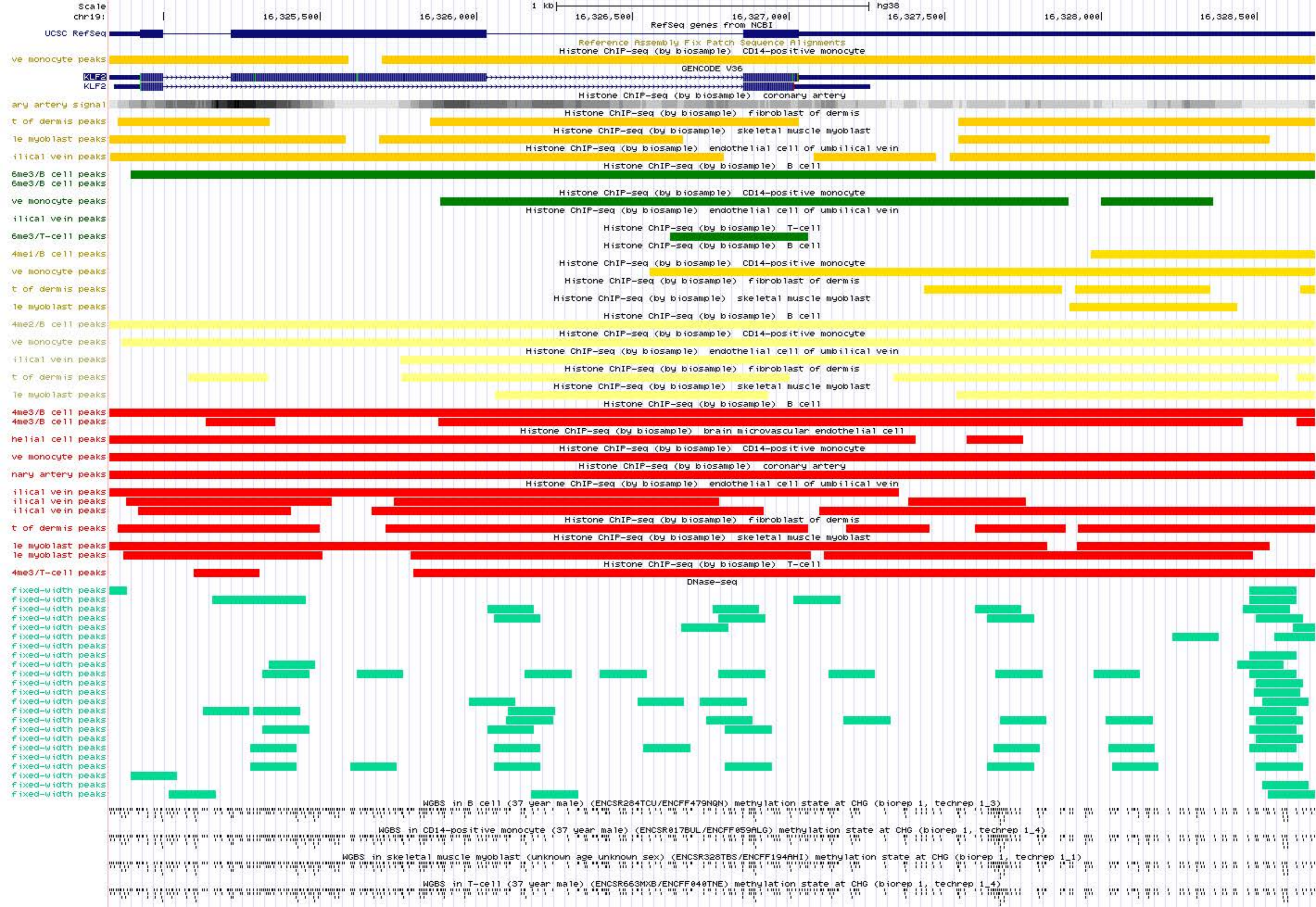

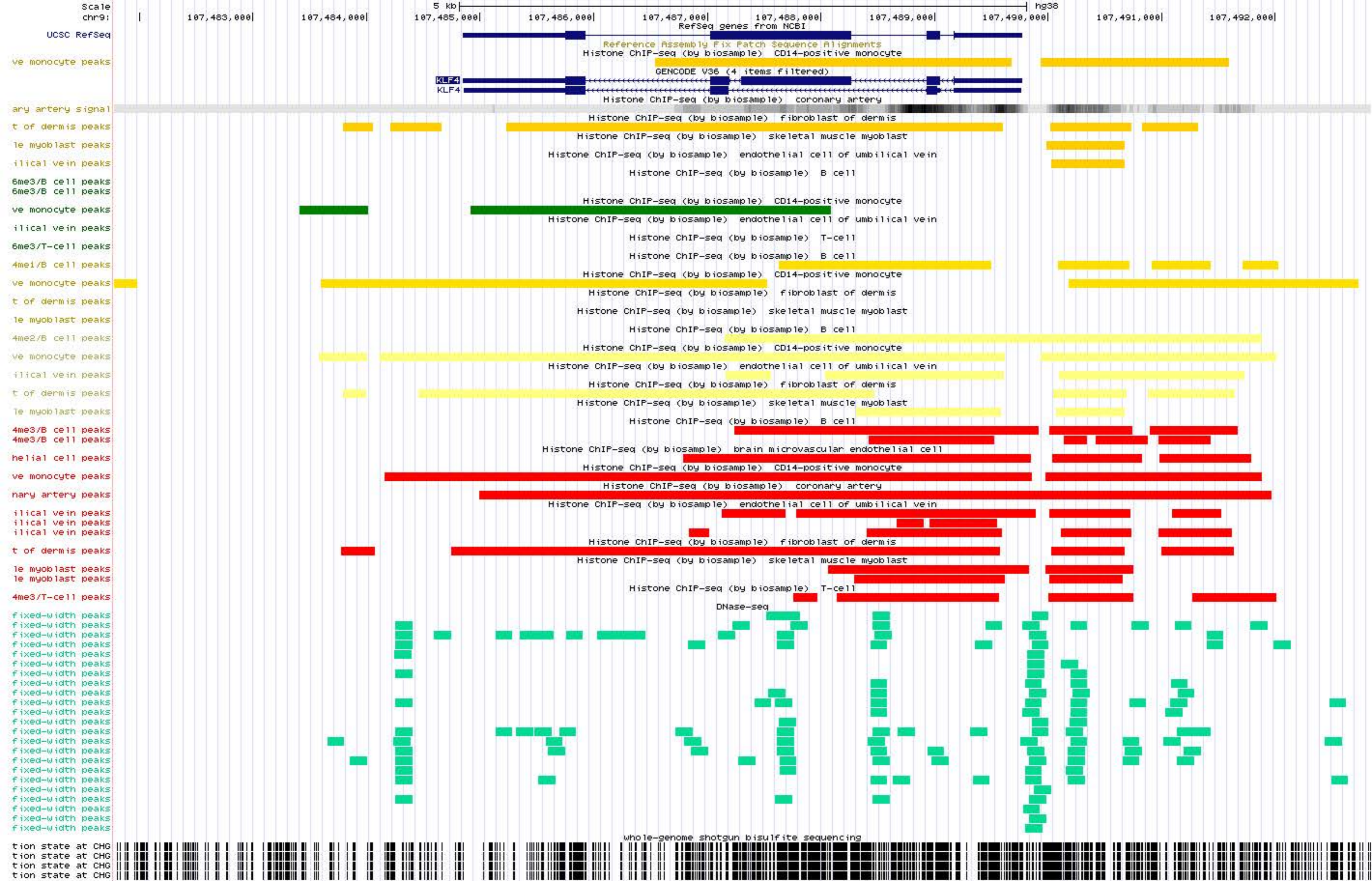

Supplement: Supplementary file 1 [file Data_Sheet_1.PDF]
